# COVID-19 pandemic: current knowledge about the role of pets and other animals in disease transmission

**DOI:** 10.1186/s12985-020-01416-9

**Published:** 2020-10-02

**Authors:** Mulugeta Kiros, Henok Andualem, Teklehaimanot Kiros, Wasihun Hailemichael, Sisay Getu, Alene Geteneh, Derbie Alemu, Woldaregay Erku Abegaz

**Affiliations:** 1 Department of Medical Laboratory Sciences, College of Medicine and Health Sciences, Debre Tabor University, Debre Tabor, Ethiopia; 2 Department of Medical Laboratory Sciences, College of Health Sciences, Woldia University, Woldia, Ethiopia; 3 Department of Medical Laboratory Sciences, Arba Minch College of Health Sciences, Arba Minch, Ethiopia; 4 Department of Microbiology, Parasitology, and Immunology, School of Medicine, Addis Ababa University, Addis Ababa, Ethiopia

**Keywords:** COVID-19, SARS-CoV-2, Pets, Domestic animals, Wild animals, One health, Animal model

## Abstract

On 11 March 2020, the World Health Organization (WHO) announced Corona Virus Disease (COVID-19), a disease caused by a pathogen called Severe Acute Respiratory Syndrome Coronavirus 2 (SARS-CoV-2), a pandemic. This ongoing pandemic has now been reported in 215 countries with more than 23 million confirmed cases and more than 803 thousand deaths worldwide as of August 22, 2020. Although efforts are undergoing, there is no approved vaccine or any specific antiretroviral drug to treat COVID-19 so far. It is now known that SARS-CoV-2 can affect not only humans but also pets and other domestic and wild animals, making it a one health global problem. Several published scientific evidence has shown that bats are the initial reservoir hosts of SARS-CoV-2, and pangolins are suggested as an intermediate hosts. So far, little is known concerning the role of pets and other animals in the transmission of COVID-19. Therefore, updated knowledge about the potential role of pets in the current outbreak will be of paramount importance for effective prevention and control of the disease. This review summarized the current evidence about the role of pets and other animals in the transmission of COVID-19.

## Background

Coronaviruses (CoVs) are a group of viruses that belong to the subfamily *Orthocoronavirinae* in the family *Coronaviridae*, Order *Nidovirales*. They are classified into four genera based on their genetic properties within the subfamily *Orthocoronavirinae*, namely Alphacoronavirus (α-CoV), Betacoronavirus (β-CoV), Gammacoronavirus (γ-CoV), and Deltacoronavirus (δ-CoV) [1, 2]. Both α- and β-CoV genera are known to infect mammals, whilst δ- and γ-CoVs infecting birds [1].

Seven CoVs, severe acute respiratory syndrome coronavirus 2 (SARS-CoV-2) being the seventh member of the family, have been found to infect humans and cause respiratory diseases so far. Among these, the common human CoVs (HCoVs) are HCoV-229E, HCoV-OC43, HCoV-NL63, and HCoV-HKU1, and they usually lead to common self-limited upper respiratory disease [3]. On the other hand, the recently emerged SARS-CoV and the Middle East respiratory syndrome (MERS)-CoV (including SARS-CoV-2 are responsible for atypical pneumonia [1, 4]. Both the SARS-CoV and MERS-CoV emerged in humans in 2002 and 2012 respectively and resulted in global outbreaks.

Early in December 2019, several patients with pneumonia of unknown etiology emerged in Wuhan City, Hubei Province, Central China [1]. The causative agent for this illness was later confirmed as a novel coronavirus by a laboratory and was initially named as a 2019 novel coronavirus (2019-nCoV) [5]. The World Health Organization (WHO) subsequently recommended the disease name as Corona Virus Disease (COVID-19). Meanwhile, the International Committee on Taxonomy of Viruses renamed 2019-nCoV as SARS-CoV-2 [5, 6]. WHO declared this recent ongoing viral outbreak as a pandemic on March 11, 2020, and an international public health emergency on January 30, 2020 [7]. As of August 22, 2020, more than 23 million confirmed cases and more than 803 thousand deaths have been reported worldwide, affecting 215 countries [8]. The impact of the pandemic goes beyond public health concern; it is causing devastating effects on the global economy at large.

Despite its rapid spread, there are no approved vaccines against SARS-CoV-2 nor specific therapeutic drugs so far. Thus, a better understanding of the virus's transmission vehicles is key to the overall prevention and control of this virus. In this connection, understanding animals' involvement in the transmission dynamic must be an important component of the efforts to interrupt the pace of its communicability and spread. Although so many studies are on progress, little is known so far concerning the role of pets, which are always in close contact with humans, and other animals in the spread of COVID-19 to humans. In this review, we summarized the current evidence about the role of pets and other animals in the spread of COVID-19 infection.

## Main text

### SARS-CoV-2 structure and life cycle

**Fig. 1:**
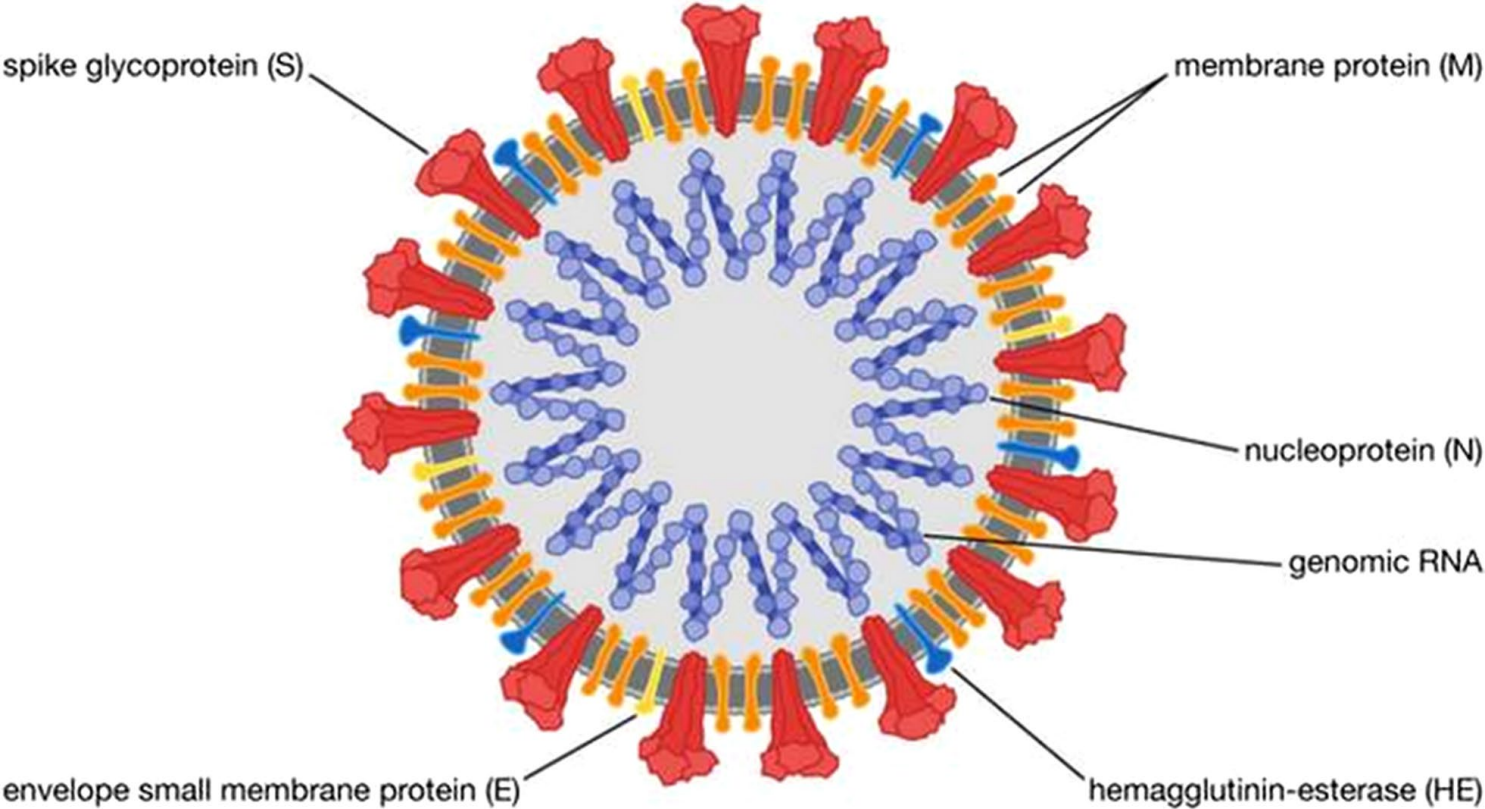
Structure of SARS-CoV-2 [17]

CoVs are enveloped viruses with icosahedral symmetry and measures ~ 80–220 nm in diameter. They are composed of a non-segmented, single-stranded positive-sense RNA genome that measures ~ 26–32 kb in size, which makes them the largest viruses among all other RNA viruses [9]. The SARS-CoV-2 is a spherical enveloped virus that measures 50–200 nm in diameter with a single-strand positive-sense RNA genome (30 kb in length) [10, 11]. The genome of SARS-CoV-2 shares 79.6% and 96% sequence identity with SARS-CoV and Bat-CoV respectively [12]. Structurally, the SARS-CoV-2 membrane contains four major structural proteins; namely, spike (S) glycoprotein, small envelope (E) glycoprotein, membrane (M) glycoprotein, and nucleocapsid (N) protein [13] (Fig. 1). S glycoprotein, which is found in the uppermost layer of the virus, mediates viral attachment to the Angiotensin-converting Enzyme 2 (ACE2) receptor on the host's target cell [14]. The M protein determines the shape of the virus is the most abundant protein than other structural proteins. Together with other structural proteins, it plays a major role in viral assembly [15]. The N protein is an RNA binding protein and as being so its main function is for binding and packaging of the viral RNA genome into a long helical nucleocapsid structure [16].

The clear picture of COVID-19 pathogenesis is still under investigation. However, in most patients, it appears to affect primarily the lung since it is a respiratory disease. SARS-CoV-2 uses the ACE2 receptor, which is found on human cells (such as in type II pneumocytes in the lungs) and other animal species for attachment [13, 18]. The binding of SARS-CoV-2 S glycoprotein to the ACE2 receptor initiates the entry pathway of SARS-CoV-2. So far, there are two proposed entry pathways [19]. In the first SARS-CoV-2 entry pathway (i.e. endosomal), the binding is followed by receptor-mediated endocytosis. An increase in H+ influx into the endosome activates cathepsin L, which then leads to the cleavage of S glycoprotein by this enzyme. This proteolytic cleavage within S glycoprotein exposes the internal fusion peptide, which is located directly adjacent to the cleavage site. Thus, upon S glycoprotein cleavage, the fusion peptide can fuse into a host cell membrane and mediates virus entry into the cell [13, 19]. The second pathway is the non-endosomal pathway, the binding of SARS-CoV-2 S glycoprotein to the ACE2 is followed by cleavage of the viral S glycoprotein by transmembrane protease serine 2 (TMRPSS2) on the surface of the host cell. This induces direct fusion of the viral and plasma membrane leading to the release of the viral particle into the cytoplasm [19]. After the virus enters the host cell and uncoats its viral genome, it is transcribed and then translated [10].

### Zoonotic origin and transmission of SARS-CoV-2

**Fig. 2:**
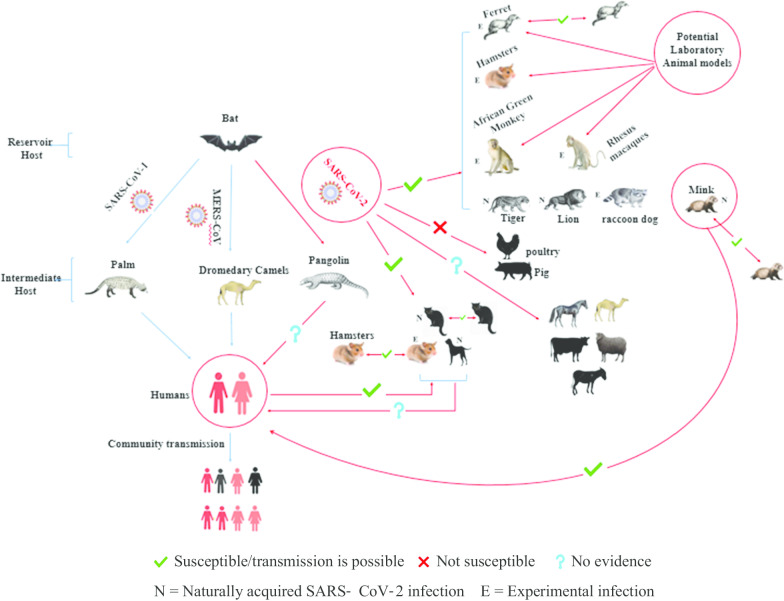
Origin and transmission of SARS-CoV-2 along with the potential role of pets and other animals in disease transmission. This figure illustrates the putative origin of CoVs and disease transmission to humans with a special focus on SARS-CoV-2. Companion animals such as cats and dogs are susceptible to SARS-CoV-2 and humans can be a source of infection for them, however, the potential role of pets in disease transmission to humans is unknown. Other animals like hamsters, African green monkeys, rhesus macaques, raccoon dogs, mink, ferret, tiger, and lion are susceptible to SARS-CoV-2 too. From these susceptible animals, hamsters, African green monkeys, rhesus macaques, and ferret are the potential animal models. Cats and hamsters from pets and mink and ferrets from other animals have the potential to transmit the virus to their respective partners. Minks can be a source of infection for humans. Given the similarity in expression of cell receptor ACE2, we assumed that it might be possible for the virus to transmit back to people again from other animals too. Hence, the usual precautionary measures should always be there as one part of a disease prevention strategy to prevent animal-animal, human to animal, and animal to human disease transmission

CoVs are known to circulate in mammals and birds [20]. According to previous studies, both SARS‐CoV and MERS‐CoV are zoonotic in origin (Fig. 2), originally coming from bats, with SARS‐CoV spreading from bats to palm civets and/or to humans, and MERS‐CoV spreading from bats to dromedary camels, and humans [21]. SARS-CoV emerged in humans that had contact with palm civets in China in 2002 and resulted in a global SARS epidemic that lasted 8 months and took 774 lives [22]. Ten years later, in 2012, MERS-CoV appeared in humans that had close contact with dromedary camels in Saudi Arabia where it remains a major public health concern, spread to 27 countries claiming 858 lives [22].

Similarly for SARS-CoV-2, due to evidence of several infected people's exposure to seafood in the wet animal market in Wuhan City, it is assumed that the virus was likely originated from animals and transmitted to humans, then maintains human-to-human transmission [23, 24]. It is now suggested that bats are the initial reservoir of the virus [25] (Fig. 2). Based on evidence from studies that investigated the animal sources of the virus pangolins are believed to be the most likely intermediate host responsible for SARS-CoV-2 human transmission [26] (Fig. 2).

Since SARS-CoV-2 primarily targets the human respiratory system, respiratory droplets and/or aerosols are considered as the main routes of transmission. Currently, COVID-19 patients remain as the primary source of infection [27], where person-to-person transmission is maintained via droplet when a person is in close contact (at least within a distance of 2 m) with an infected individual [28]. A person can also be infected through fomites in the immediate environment around the infected person, by touching infected instruments and consecutive touching of mouth, nose, and/or eyes by contaminated hand [29].

### Role of pets in disease transmission

The first COVID-19 case in companion animal was reported in a Pomeranian dog from Hong Kong, China in February 2020 [30]. Later in March 2020, COVID-19 was reported from a cat in the same country [31]. These two cases were found after their owners were reported positive for COVID-19 [30, 31]. The persistent positive reverse transcription-polymerase chain reaction (RT-PCR) result of the Pomeranian dog was accepted as a true positive by the experts from the University of Hong Kong and the World Organization for Animal Health as a true positive result for a true infection [32]. This was further supported by the absence of contamination as the dog was confined in quarantine at government kennels [32]. Genetic sequence similarities of the SARS-CoV-2 from the owner and the pets indicated the potential of human-to-animal transmission [33]. Besides, both viral culture and serological tests were done to check whether the dog was contagious or not and becomes negative. These tests coupled with the absence of any sign and symptom lead to deduce that the dog was not contagious to humans and/or another animal [32].

After the first reported case of COVID-19 in a cat, other COVID-19 cases were similarly reported from other countries like Belgium [34], France [35], Germany [36], Russia [37], and the United States [38]. Based on these reports and other evidence from experimental studies, it is now accepted that these two companion animals are susceptible to SARS-CoV-2 with cats being highly susceptible and having the potential to transmit the illness to other naive cats while dogs are less susceptible [39]. Besides cats and dogs, the golden Syrian hamsters have been also confirmed to be susceptible to SARS-CoV-2 in recent laboratory experiments [40, 41]. It was demonstrated that golden hamsters exposed to SARS-CoV-2 can be infected and were able to efficiently transmit the virus to naive hamsters by direct contact and via aerosols [40].

Altogether, pets living in the household of people with COVID-19 are at risk of contracting the disease and can spread the virus to other naive pets. Hence, owners should protect their companion animals from being infected, which will have a positive effect on COVID-19 prevention. Unless pet owners do so, this would pose difficulties for the overall prevention and control of the disease. However, there is currently no evidence on the potential role of pets on SARS-CoV-2 transmission to humans. Although there is no evidence, these pets might have the potential to transmit the virus to humans as they express the same cell receptor ACE2 [15]. Hence, the usual precautionary measures must always be there.

### Role of domestic and wild animals in disease transmission

Similar to the susceptibility of the aforementioned companion animals, other domestic animals such as ferrets were also reported to be highly susceptible to SARS-CoV-2 [39]. A recent experimental study verified that infected ferrets can transmit SARS-CoV-2 efficiently to other naive ferrets via direct contact and air [42]. In contrast, other domestic animals like pigs and poultry are not susceptible to SARS-CoV-2 [39, 43] while there is no evidence so far on the susceptibility of livestock animals like camel, horse, sheep, cow, and donkey. In another recent experimental study, raccoon dogs were also found to be permissive to SARS-CoV-2 and were reported to have the potential to transmit the virus to other contact animals. According to this study, high viral replication was observed in the upper and lower respiratory tract following intranasal inoculation of SARS-CoV-2 [44].

Concerning other wild animals, tiger and lion were confirmed to be susceptible to SARS-CoV-2 [45, 46]. In April 2020, five tigers (Two Malayan and three Amur tigers) and three African lions that exhibited respiratory signs (dry cough and some wheezing) were tested positive in the Bronx Zoo in New York City, USA. It is assumed that an asymptomatic zoo employee infected the animals [45, 46]. Minks, which are farmed for their fur, are also susceptible to SARS-CoV-2 with the ability to transmit the virus among each other [47]. The first positive mink case was reported from the Netherlands on 23 and 25 April, where there are around 125 mink farms. Some of the workers at the farm had previously tested positive for the SARS-CoV-2; therefore, it is assumed that human-to-animal transmission was the most likely scenario for the infection of the minks [47]. Since the above-mentioned first reported case, other countries like Denmark [48] and Spain [49] have also reported similar COVID-19 cases in this animal. These all are additional evidence, which indicates that human to animal transmission is possible. Hence, people who are in contact with these animals and/or work on wildlife conservations should follow strict measures. This will have a negative impact on animal farms and/or wildlife unless the necessary preventive measures are taken. Other animals from which SARS-CoV-2 was detected include Egyptian fruit bats African green monkeys and rhesus macaques; suggesting the possibility of cross-species transmission of SARS-CoV-2, although observed rarely [43, 50–52].

A recent research finding from the Netherlands suggested that there has been a transmission of a new case of COVID-19 from mink back to human (an employee who worked in a mink farm). This was supported by viral sequence similarities between an infected employee and the virus found from a mink on the same farm, leading the researchers to conclude that one employee the mink farm was infected by the virus transmitted from an infected mink with no apparent disease manifestation [47]. This study has also shown that COVID-19 infection in mink can be asymptomatic which indicates the possibility of mink as an intermediate host. Since it has been described that except rats and mouse a range of species express the cell receptor ACE2 [15], it should also be noted that animal-to-animal and animal-to-human transmission is possible in addition to human-to-human [15]. This might be the case of the mink from the Netherlands [47].

### Laboratory animal models

Laboratory animal models that mimic human disease are very crucial to understand the pathogenicity of a given disease and investigate vaccine candidates and potential therapies. Previously, non-human primates, mice, and hamsters that showed similar signs and symptoms of infection and viral replication with humans were used as animal models to study SARS-CoV and MERS-CoV [53, 54]. Similarly, various small animals and non-human primates are proposed as a preferred animal model for studying SARS-CoV-2.

The golden Syrian hamsters are one of the proposed laboratory models that can be used to study SARS-CoV-2 as it showed apparent signs like weight loss and an efficient viral replication in the nasal mucosa and epithelial cells of the lower respiratory system [40, 41] (Fig. 2). Similarly, ferrets, which have been frequently used as an animal model for other human respiratory viruses [55, 56], are also proposed to be used as a potential animal model to study SARS-CoV-2 [39]. In an experimental infection, SARS-CoV-2 was able to replicate efficiently in the upper respiratory tract of this animal for up to eight days without causing severe disease or death [39].

Other recent studies have examined the utility of non-human primates, rhesus macaque, as a model for carrying out SARS-CoV-2 studies [50, 51]. This macaque, which has been shown to be infected and presented with a sign and symptom and viral shedding similar to humans, is now considered as of the preferred potential laboratory animal models for SARS-CoV-2 (Fig. 2) [50, 51]. In another similar experimental study, African green monkeys were also reported to be a promising animal model for studying COVID-19 infection as they mimic human conditions upon viral inoculation [52].

### Laboratory diagnosis of COVID-19 in animal

Laboratory diagnosis of SARS-CoV-2 in animals is similar to the viral diagnosis among humans. For diagnosis, respiratory tract specimens from the nasal turbinate, soft palate, and tonsils are preferred [39]. Although specimens from these sites are preferable, other specimens from other sites like rectal swabs may also be used in situations where direct sampling is not possible due to risks to the animal or testing staff [57]. The molecular test developed for use in human samples; the real-time Reverse-transcription polymerase chain reaction (RT-PCR), is the gold standard and widely used method to diagnose SARS-CoV-2 in animals using the above-mentioned samples [42, 58]. Besides, viral isolation in cell culture, viral genome sequencing, and other molecular tests like Reverse transcription loop-mediated isothermal amplification (RT-LAMP) can also be used for the detection of SARS-CoV-2 in animals [57]. Furthermore, rapid immunochromatographic tests and other serological immunoassays such as enzyme-linked immunosorbent assay (ELISA) and Virus neutralization tests can be also used for the detection of antibodies against SARS-CoV-2 in animals [57, 59].

### Prevention and control

Although many vaccines and antiviral drugs are being tested, there is no known effective treatment for COVID-19 so far. Effective prevention and control strategy is primarily through the mitigation of human-to-human spread. This includes personal protection (like personnel hygiene, wearing a facemask), social distancing, temperature screening, early testing, quarantine of peoples of suspected or infected individuals' travel history, and preventing further global spread [60–62]. Since studies have shown that the origin of the virus is connected to a seafood market in Wuhan, identification and reduction of transmission from the susceptible animal sources might also another means for prevention and control of viral spread [63, 64]. Moreover, social distancing, use of personal protective equipment (PPE) by health professionals, and persistent use of facemasks by the general population, quarantine, and surveillance are still considered as the most effective means of controlling the human-to-human spread. As part of the surveillance measures, conducting temperature screening, early testing, and isolation of peoples of suspected or infected individuals and those with travel history are essential preventive actions [60].

Given the susceptibility of pets and other animals to SARS-CoV-2, it is still recommended that people who are suspected or confirmed for COVID-19 should limit contact with these animals as this will minimize animal infection from human sources [65]. Similar to the case of the Mink from the Netherlands, companion animals may also have the potential to spread COVID-19 to other people in the household or people being in close contact with the animals. It is thus advisable that humans to avoid unnecessary contact with animals and care, like basic hygiene measures, should always be there when handling and caring for animals and/or animal products [65]. Additionally, animals belonging to owners infected with SARS-CoV-2 should be kept indoors in line with similar lockdown recommendations for humans to prevent animal-to-animal spread [65]. Overall, countries should have a one-health approach in their prevention and control strategy to protect both humans and animals from being infected, which can have a positive impact on prevention and control and consecutively in the economy.

## Conclusion and future perspective

Taking the rapid spread of COVID-19 across the globe into account, here we reviewed the latest updates about SARS-CoV-2 with special emphasis on the role of pets and other animals on disease transmission, which will have implications for prevention and control of the disease. Animals could play an important role in SARS-CoV-2 disease transmission. The recently confirmed COVID-19 cases in the Netherlands due to mink could suggest that animal to human viral spillover is possible. In the current SARS-CoV-2 pandemic, the situation is rapidly evolving and in light of the recent evidence, we should be aware of the possibility that humans can be potentially infected with COVID-19 by animals, including pets or other domesticated species. Therefore, the usual precautionary measures should always be there as one part of a disease prevention strategy when dealing or spending time with companion animals. Tracing of SARS-CoV-2 infection of pets especially for those whose owners are positive and consecutive isolation would also be important for preventing the resurgence of COVID-19. Unwanted abandonment of companion animals should be avoided. Rather tracing of infected animals and surveillance should be there to prevent further transmission. Along with other successful management of the pandemic, it is very critical to conduct further studies on the overall zoonotic risks of SARS-CoV-2 and on the possible intermediate host to prevent the re-emergence of the virus. Furthermore, we recommend all countries enhance their strategy for animal epidemic prevention and control like the Netherlands, Spain, USA, and China (one health approach).

## Data Availability

Not applicable.

## Abbreviations

ACE2: Angiotensin-converting enzyme 2
CoVs: Coronaviruses
COVID-19: Coronavirus disease 2019
HCoV: Human coronavirus
MERS: Middle East respiratory syndrome-related coronavirus
SARS-CoV-2: Severe acute respiratory syndrome coronavirus 2
WHO: World Health Organization
